# Improving quality of teaching and child development: A randomised controlled trial of the leadership for learning intervention in preschools

**DOI:** 10.3389/fpsyg.2022.1092284

**Published:** 2023-01-04

**Authors:** Iram Siraj, Edward Melhuish, Steven J. Howard, Cathrine Neilsen-Hewett, Denise Kingston, Marc De Rosnay, Runke Huang, Julian Gardiner, Betty Luu

**Affiliations:** ^1^Department of Education, University of Oxford, Oxford, United Kingdom; ^2^Early Start and School of Education, University of Wollongong, Wollongong, NSW, Australia

**Keywords:** early childhood, professional development, intervention, randomised controlled trial, quality rating scales, child development

## Abstract

**Introduction:**

Substantial research indicates that high quality early childhood education and care (ECEC) confers a wide range of benefits for children, yet quality in ECEC remains inconsistent. Given the variability in training and qualifications, one strategy for improving ECEC quality is in-service professional development (PD).

**Methods:**

The current study evaluated an evidence-based in-service PD programme, Leadership for Learning, via a cluster randomised controlled trial involving 83 ECEC services and 1,346 children in their final year of pre-school.

**Results:**

Results indicated significant improvements in teaching quality across treatment centres and child development outcomes in language, numeracy and social-emotional development.

**Discussion:**

This study provides strong support for making evidence-informed PD routinely available for ECEC practitioners.

## Introduction

A growing number of studies that examine the association between early childhood education and care (ECEC) and children’s developmental outcomes have demonstrated that children who attended preschools tended to show better early academic attainment and social–emotional wellbeing than those who did not attend (Sylva et al., 2004, 2014; Melhuish et al., 2015; Lehrl et al., 2016). The positive effects of ECEC provision on individuals have also been shown to last into adolescence (Sylva et al., 2014). Quality is important, yet there is variability in this across the sector including in teachers’ characteristics, classroom and preschool structural features and social-cultural contexts (Alexandersen et al., 2021).

Many staff, therefore, do not possess the necessary skills and knowledge to support children’s effective learning in ECEC programmes (Howes et al., 2008). Their lack of practical and theoretical knowledge of how children develop and learn renders them unable to justify their practice, promote children’s learning and defend their own professionalism (Stephen, 2012). Consequently, increasing attention has been focused on teacher professional development (PD), which might improve teachers’ instructional quality and thereby impact children’s learning and development (Darling-Hammond et al., 2017; Egert et al., 2018).

A variety of in-service PD approaches—such as lectures, workshops, coaching, mentoring and professional learning communities—have been advocated to improve teaching and learning (OECD, 2016); however, most of them have been limited to a specific learning area. Relatively little is known about the effectiveness of comprehensive PD programmes (Egert et al., 2018). Accordingly, this study aimed to implement and evaluate an evidence-based, in-service PD programme to provide sustainable, practical, relevant support for preschool staff, and to improve teachers’ pedagogical quality and children’s developmental outcomes using quality rating scales supported by training through PD workshops.

## Literature review

### Combining relational and intentional pedagogies for better child development

Increasing research has demonstrated that the *process* quality of ECEC is an important predictor of early childhood development (Hatfield et al., 2016; Siraj et al., 2018). Effective pedagogical practice, which include sensitive teacher-child interactions around curricula content in a positive climate, is a key element of process quality (Howes et al., 2008). This definition of effective pedagogical practice aligns with Kingston and Siraj (2017) work, which explores teachers’ pedagogical practice from two perspectives: relational and intentional pedagogies. The former refers to teachers’ beliefs and actions in building an emotional and individual relationship with children; while the latter focuses on teachers’ knowledge and intention to help children develop knowledge, skills and dispositions. Effective teachers integrate positive relationships with educational intention and combine intentional instruction with warm and respectful interactions (Howes and Tsao, 2012; Kingston and Siraj, 2017).

In order to explore how pedagogical practice works on child development, Nguyen et al. (2020) conducted a large-scale investigation that involved 1,498 children from 156 classrooms from varied cultural and linguistic backgrounds. They found that teacher-child relationships and intentional pedagogy worked independently and synergistically to promote children’s academic, cognitive, and social–emotional outcomes. For one thing, continuing warm, respectful and supportive teacher-child relationships are the foundation of effective pedagogy—as scaffolding child development within children’s zone of proximal development (ZPD) is based on teachers’ sound understanding of children’s individual characteristics, e.g., learning style, interests and preferred learning areas (Vygotsky, 1978; Kingston and Siraj, 2017). Further, within positive relationships, effective pedagogy also requires high-quality intentional pedagogical interactions which aim to scaffold children’s learning and thinking (Siraj and Kingston, 2015). When teachers model language use, scaffold children’s conceptual understanding and provide feedback within pedagogical interactions, they influence children’s language skills, thinking and early academics (Hatfield et al., 2016). In addition, the combination of relational and intentional pedagogies enables responsible and responsive practices that not only meet children’s needs but also purposefully develops children’s minds in different socio-cultural contexts. It aligns with competence models that require teachers to support children’s knowledge, skills and attitudes corresponding to the context, such as personal fulfilment, social inclusion and citizenship (Urban et al., 2012).

This construct of pedagogical quality is supported by attachment theory (Bowlby, 1969) and social constructivist theory (Vygotsky, 1978). According to the attachment theory, children with a secure relationship with their caregivers will be open to using their caregivers’ help to develop skills. In classrooms, teachers act as alternative caregivers who can promote children’s development by fostering positive teacher-child relationships. Meanwhile, the social constructivist theory (Vygotsky, 1978) regards knowledge as constructed through interacting with (more knowledgeable) others. Knowledge is built when individuals experience progressively more complex interactions with teachers (or peers). Accordingly, it could be argued that PD programmes seeking to enhance effective teaching and learning should combine relational and intentional pedagogies to improve teacher-child interactions and child-developmental outcomes.

### Promoting pedagogical quality and child development through professional development programmes

Professional development is a process involving teachers learning and then applying what they have learnt to practice—to support children’s learning (Avalos, 2011). High quality and continuing PD can supplement teachers’ theoretical learning (usually obtained through their formal education) and equip them with newly-adapted knowledge and strategies which are more relevant to real teaching practices (OECD, 2012; Manning et al., 2019). Participating in effective PD programmes that can promote teachers’ professional knowledge, skills and attitudes is the primary approach to in-service teachers’ learning (Fukkink and Lont, 2007; Siraj et al., 2018).

Diverse PD programmes (e.g., workshops, coaching and professional learning communities) have been designed to develop teachers’ knowledge base of child development and teacher-child interaction for the enhancement of their pedagogical practices (e.g., Burchinal et al., 2002; Buysse et al., 2010; Sedova et al., 2016; Early et al., 2017; Gore et al., 2017). Recent meta-analyses on in-service PD and child development demonstrated that improvement of pedagogical quality was related to children’s developmental outcomes and that enhancing pedagogical quality was the key mechanism for promoting child development (Werner et al., 2015; Egert et al., 2018). Relational and intentional pedagogy are two distinct foci of current PD programmes targeting enhancing pedagogical quality and benefiting child development.

Some programmes emphasise relational pedagogy and aim to promote teacher-child relationships and children’s social–emotional development by training teachers to provide responsive and supportive interactions (e.g., Sandilos et al., 2018; Rudasill et al., 2020). For example, Rudasill et al. (2020) conducted a PD intervention to promote higher quality teacher-child relationships by improving teachers’ understanding of temperament. They found that the improved teacher-child relationship facilitated children’s social–emotional and self-regulation skills. Sandilos et al. (2018) also observed that reduced professional stress after PD could predict teachers’ higher emotional support for children. Teachers who have attended training related to teacher-child relationships and emotional support tend to conduct more child-sensitive, emotionally supportive learning environments to meet children’s interests and needs. Relational pedagogy helps staff to interact with young children in a shared, stimulating and meaningful manner (Siraj-Blatchford et al., 2002; Ansari and Pianta, 2018).

Meanwhile, McCoy and Wolf (2018) found that improvements in instructional aspects of classroom quality predicted children’s academic *and* social–emotional gains, while the social–emotional dimensions of quality did not. Therefore, some programmes emphasise intentional pedagogy and aim to promote instructional support and children’s academic development by providing higher quality learning-oriented interactions (e.g., Sedova et al., 2016; Gore et al., 2017). For example, Sedova et al. (2016) provided knowledge, learning activities and examples of teaching practice to promote teachers’ dialogic teaching through workshops, documentation and reflective interviews. Teachers used more high-cognitive, challenging questions and open discussions to foster students’ reasoning after the PD. Wasik and Hindman (2018) promoted teachers’ instructional quality and use of vocabulary strategies and children’s vocabulary development by implementing a book-reading intervention for teachers. PD programmes focusing on intentional pedagogy help teachers obtain practical teaching knowledge *and* provide high-quality instructional support for children’s learning.

Therefore, most research on the effects of PD related to child outcomes focuses on a particular domain, rather than taking a holistic perspective of teachers’ pedagogical practice and child development. A relatively comprehensive perspective to integrate intentional and relational pedagogy could be employed. Besides, specialised PD cannot always guarantee its effectiveness on child development due to its diverse content, different expertise of tutors and the varying training organisation or structure (Siraj et al., 2018). Buysse et al. (2010) reported significant improvement in teachers’ teaching practices but minimal effect on child development. The mixed and inconclusive findings warrant further research to integrate the teacher-child relationship with pedagogical interaction and to examine whether and how PD programmes may influence children’s learning and development.

### The study programme of *leadership for learning*: An evidence-based professional development programme

To identify the key requirements for designing effective PD programmes, Buysse et al. (2009) identified three critical components of effective PD programmes: the “who,” “what,” and “how” of PD intervention. “Who” refers to the receivers, providers and context of a PD programme; “what” focuses on the content of PD—such as the targeted knowledge, skills and attitudes that the PD programme aims to achieve and ‘how’ includes the duration, training approaches and training formats. Based on Buysse’s model, Egert et al. (2018) conducted a meta-analysis to analyse three components of PD and found different combinations of the components generated different results of PD. This framework was used to develop and define the components of this study’s *Leadership for Learning* PD programme.

Furthermore, the *Leadership for Learning* programme was also informed by the findings from empirical evidence in effective PD and high-quality ECEC provision. It aims to improve the quality of teaching and learning by preparing teachers for a leadership role within their classrooms and preschools. Empowering teachers to take leadership roles has powerful influences on teachers’ motivation and self-esteem, which leads to a higher retention rate and quality of teaching (Muijs and Harris, 2003). Leadership emerges when teachers attain strong pedagogical and content knowledge, collaborative skills and the ability to influence their colleagues (Snell and Swanson, 2000). These elements foster teachers’ leadership and have been included in the “Leadership for Learning” PD programme. It aligns with the framework of PD programmes showing these distinctive features of PD in the extant literature. Zaslow et al. (2010) collated previous research which had shown a number of other potential influences on the quality of PD practice, and identified six features of more-effective programmes:

- *Clear*, *articulated objectives for the PD*.
- *Explicit focus on practice in the PD*, *based on staff knowledge and practice*.
- *Collective participation by staff from the same settings*.
- *Intensity and duration of the PD matched the content*.
- *Staff prepared to engage in assessments and interpret their results as a tool for ongoing monitoring of the effects of the PD*.
- *Appropriate for the setting context and aligned with standards for practice*, p. xii-xiv.

Darling-Hammond et al. (2017) also stressed the importance of content, active learning, modelling effective practice, coaching, and feedback: many of these dimensions are implicit or explicit in a range of the extant research literature. Taking these elements into account, the Leadership for Learning programme was developed in the Australian ECEC context. In Australia, the National Quality Framework (NQF) was implemented by the Children’s Education and Care Quality Authority (Australian Children’s Educational and Care Quality Authority, 2018) to rate ECEC services by four levels: needing significant improvement; working towards National Quality Standards (NQS); meeting NQS and exceeding NQS. There is a fifth excellent rating category which has to be applied for, but it is very rare (Siraj et al., 2018). The Leadership for Learning programme was constructed against this background to provide PD for early childhood programmes with a variety of quality ratings. Teachers from these early childhood programmes were given the opportunity to receive the PD.

Regarding the “what” component, the programme was developed with close reference to recognised quality rating scales the Early Childhood Environmental Rating Scale–Extension (ECERS-E) and the Sustained Shared Thinking and Emotional Wellbeing (SSTEW), and the NQS (Siraj et al., 2018). Previous studies have shown that PD with a specific focus is more effective than PD with a generic focus on pedagogy (Buysse et al., 2009), and PD, which focuses on improving relational and intentional teacher-child interaction, is especially effective at improving teachers’ quality of teaching (Fukkink and Lont, 2007). Given these findings, the Leadership for Learning programme tailored its PD content to focus on measuring the quality of the curriculum, interaction in relational and intentional pedagogy—as well as children’s cognitive and social–emotional development—to meet the preschools needs and contexts.

Regarding the “how” component, the Leadership for Learning programme used different types of PD approaches to equip teachers with skills and knowledge related to teacher-child interaction, child assessment and pedagogy through on-site modelling, providing DVD exemplars and guided deconstruction of high-quality interaction, and providing reading materials as well as training on the use of the two quality rating scales for in-centre practice improvement (Siraj et al., 2018).

These approaches are supported by existing literature. For instance, face-to-face training has been shown to generate enhanced teacher-child interactions and improved language, social and physical development amongst children (Pianta et al., 2008; Downer et al., 2009). Teachers tend to implement improved instructional practices when they receive mentoring and coaching in PD (Gore et al., 2017; Kraft et al., 2018). And, based on the findings of their meta-analysis, Egert et al. (2018) suggest that a 45–60 h PD course is more effective than shorter or longer programmes.

On this basis, Leadership for Learning consists of 2-days’ intensive face-to-face training, five 4-h face-to-face workshops, and follow-up web-based intervention to ensure and sustain the training effects. To bridge the gap between theories and teaching practice, the programme provides training sessions to update teachers’ knowledge of high-quality teaching, takes teaching guidance to teachers’ real workplaces, segments teachers’ changes into continuous phases and encourages teachers’ reflections (Sedova et al., 2016).

For example, in the adaption phase, more opportunities and individualised feedback are provided for teachers to practice what they have learned from trainers, and to reflect on their implementation (Joyce and Showers, 2002; Kraft et al., 2018). After each training phase, teacher participants are invited to participate in individual interviews and to complete questionnaires to gauge their reflections and understand their needs in the next stage.

Overall, this study aims to evaluate whether the Leadership for Learning PD programme can generate substantial and practical improvements in teachers’ pedagogical quality—and, hence, child outcomes—in one state in Australia. The training and use of two quality rating scales that teachers can refer to and self-rate on also aims to democratise assessment for improvement.

### Theoretical frameworks for analysing the effects of professional development programmes

Some previous studies have attempted to understand how PD programmes may influence teachers’ knowledge, their practice and children’s development (e.g., Harris, 1980; Guskey, 1986; Clarke and Hollingsworth, 2002; Egert et al., 2018). For example, Clarke and Hollingsworth (2002) constructed a comprehensive model, *the interconnected model of teacher professional growth*, which involves changes in (i) personal attributes (changes in knowledge, belief and attitude); (ii) professional practice (changes in professional experimentation); (iii) direct consequences (changes in students’ learning motivation and learning outcomes, and teachers’ pedagogical practice); and (iv) external context (changes in information and stimuli, such as relevant readings and conversations).

The underlying mechanism aligns with the theory of change (TOC), which can be used to examine whether, how and why a PD programme works/fails. TOC specifies how initiatives generate desired outcomes and the contextual conditions which influence the process (Connell and Kubisch, 1998). According to TOC, a programme can lead to early, interim and long-term outcomes, while the earlier changes can precede the interim and long-terms ones (Connell and Kubisch, 1998). As indicated by Clarke and Hollingsworth (2002), child development is the ultimate goal, which can be realised after teachers improve their knowledge and pedagogical practice *via* PD programmes. Markussen-Brown et al. (2017) employed TOC to indicate that PD could improve children’s development as teachers’ training outcomes would lead to improved structural features of classrooms (e.g., the provision of learning materials and establishment of targeted learning centres) and process quality (e.g., instructional strategies and teacher-child interaction).

At the same time, contextual factors also impact PD programmes and have the potential to achieve the desired outcomes (Connell and Kubisch, 1998). Various individual and social–cultural, and structural factors might influence teachers’ willingness to participate in or apply what they have learned into practice, thus affecting PD effectiveness (McConnell, 2022). For example, teacher-child interaction differs between boys and girls and girls tend to receive more positive attention than boys, which moderates the PD effects (Consuegra and Engels, 2016). Considering the aforementioned PD impacts and personal and contextual factors, TOC has been employed in this research to guide the analysis of PD impacts on pedagogical quality, child development and possible influencing factors. In particular, it was guided by the following research questions:

1. What are the impacts of the *Leadership for Learning* PD on pedagogical quality?
2. What are the impacts of the *Leadership for Learning* PD on children’s language and numeracy skills?
3. What factors affect the impacts of the *Leadership for Learning* PD on child development?

## Materials and methods

### Study design

The Fostering Effective Early Learning (FEEL) study—pre-registered with Australian New Zealand Clinical Trials Registry (ACTRN12616000536460) and protocols published in advance (Melhuish et al., 2016)—employed a cluster randomised controlled trial (RCT) design. Ninety ECEC centres in metropolitan and regional areas in NSW, Australia, were recruited to ensure representation across government quality ratings, geography, service type and socioeconomic area. The study recruited classrooms in the year before school entry.

**Figure 1:**
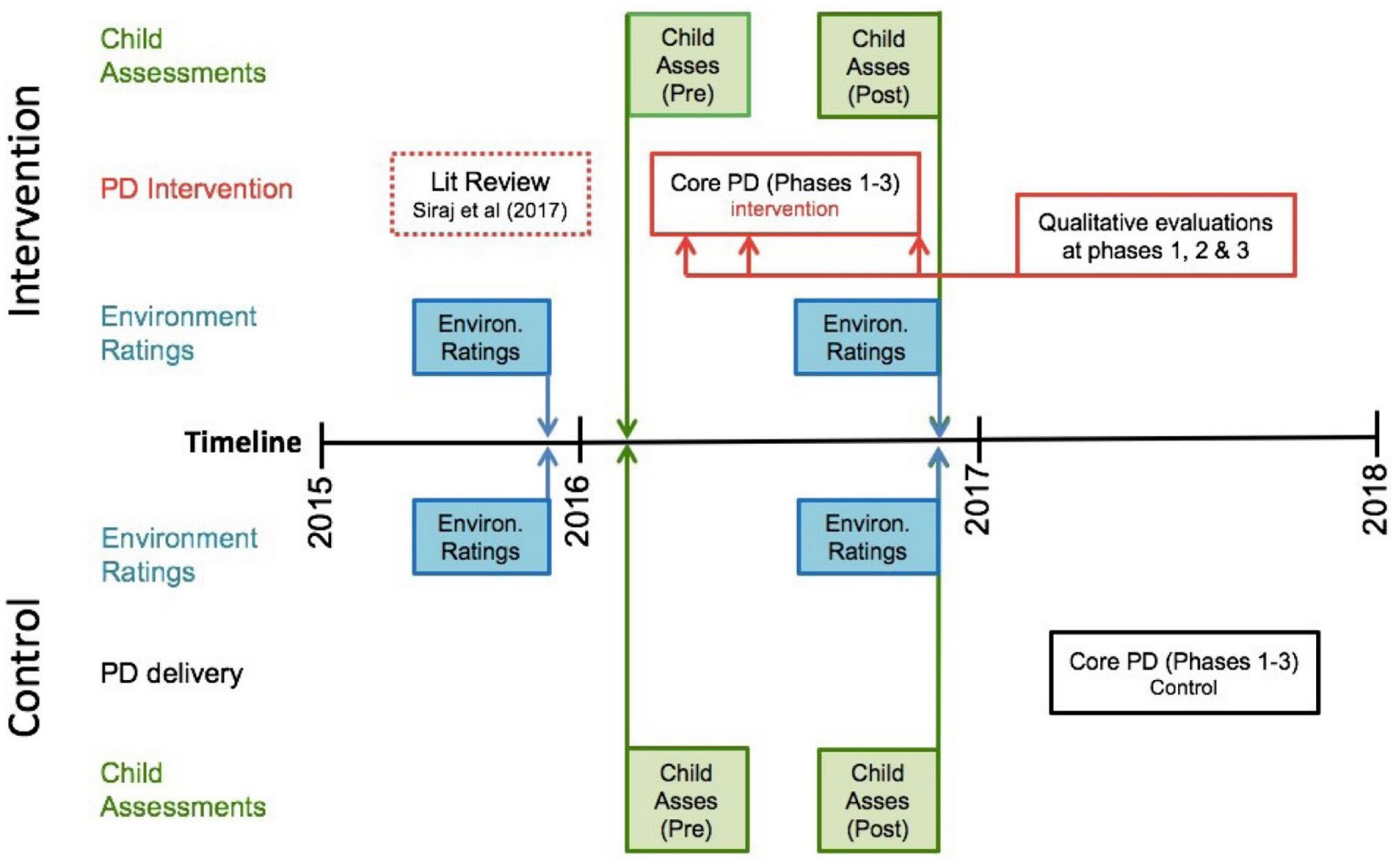
The design of the cluster RCT examines the efficacy of the leadership for learning professional development.

The sequence of the study is depicted in Figure 1. After centre recruitment, quality ratings were conducted at the end of the year prior to the PD intervention programme. This was to ensure quality ratings were taken at the same time of year—when the educators were most familiar with the children—both before and after the intervention. Centres were then randomly assigned to one of two groups: (a) an intervention group (*n* = 45 centres) which would receive the PD intervention and (2) a control group (*n* = 45 centres) which would continue engaging in typical classroom practice. Data collectors, blinded to group allocation, conducted baseline child assessments early in the intervention year. Post-intervention child assessments and quality ratings were conducted at the end of the 7-month intervention period. Ethical standards were followed rigorously *via* university ethics committee and regular consultation with funders and researchers. Centres, educators and parents were provided written consent as a condition of participation, and children provided their verbal assent.

### Participants

#### Centre characteristics

Ninety ECEC centres were recruited from areas surrounding one metropolitan hub (*n* = 45) and two regional hubs (*n* = 45) in Australia. These were largely balanced in geographic location (42 regional, 49 metropolitan) and National Quality Standard (NQS) ratings (25 *working towards*, 27 *meeting*, 37 *exceeding*, 2 *not yet rated*). The centres were intentionally unbalanced in service type (64 long-day care, 27 preschool), to mirror the sector in the state (Australian Bureau of Statistics (ABS), 2016b). Disadvantaged areas were deliberately oversampled (46% from deciles 1–3, 54% from deciles 4–8, based on the Australian Bureau of Statistics’ Socioeconomic Indexes for Areas, or SEIFA; Australian Bureau of Statistics (ABS), 2016a).

After the baseline ECERS-E (Sylva, 2010) and SSTEW (Siraj et al., 2018) quality ratings had been completed in each participating classroom, centres were randomly assigned to the intervention or control group. Following this, seven centres assigned to the intervention group (17%) withdrew from the study because they did not have the capacity to attend the PD: two had maternity leave for key staff and five had key staff resign, which is typical of staff turnover in the sector (United Voice, 2014). All dropouts occurred before commencing the PD, resulting in an intervention group containing 38 ECEC centres: the final sample’s characteristics are presented in Table 1.

**Table 1:** Final sample centre characteristics by group.

	Intervention	Control
Number of centres	38	45
# of pre-school rooms	39	54
Geographic location	18 regional, 20 metro	18 regional, 27 metro
Service type	28 long-day care, 10 preschools	31 long-day care, 14 preschools
NQS rating	9 WT, 9 M, 19 E, 1 UR	12 WT, 14 M, 18 E, 1 UR
SEIFA decile	*M* = 3.84 (45% decile 1–3)	*M* = 3.89 (49% decile 1–3)

NQS refers to statutory National Quality Standard, against which centres are rated. Ratings refer to working towards (WT), meeting (M), exceeding (EX), or unrated (UR) against this Standard at the time of recruitment. SEIFA is an area-level socioeconomic status index, given here as decile, developed by Australian Bureau of Statistics (ABS) (2016a).

#### Child characteristics

The final sample comprised 1,346 children aged 4 to 5 years, with an average cluster size of 14 per room with whom child assessments were conducted (see Table 2). This corresponded to a consent rate of 57% amongst those invited to participate and a participation rate of 96% amongst consented children. Non-participation was due to absence at time of assessment (*n* = 56 children) or early withdrawal from the centre (*n* = 8 children). The final sample had an average age of 4.59 years at baseline (*SD* = 0.37; range: 3.10–5.69 years) and a slight over-representation of boys (*n* = 735, 55%). Available socio-demographic data (reported for 96% of children) indicated that parents were born predominantly in Australia (87%), were English-speaking at home (90%), and had a range of maternal education levels (42% with a degree or higher, 18% with a diploma or certificate, 40% completed high school) and family income (20% low, 46% middle and 34% high, as defined by Australia’s Defined Child Benefit income thresholds). Children identified as Aboriginal or Torres Strait Islander (4%) were slightly under-represented relative to the general population (6%; Australian Bureau of Statistics (ABS), 2011). As shown in Tables 2, 3 there was no significant difference in child characteristics between the intervention and control groups.

**Table 2:** Child characteristics in intervention/control group.

Variable	Level	Control		Intervention		value of p
		*N*	%	*N*	%	
Sex	Male	372	55.6	363	53.6	0.498
	Female	297	44.4	314	46.4	0.498
Mother’s education	Less than high school	66	10.2	79	12.3	0.273
	High school or equivalent	188	29.0	189	29.3	0.943
	Diploma	122	18.8	110	17.1	0.456
	University or higher	272	42.0	266	41.3	0.851
Income band	Low	169	29.4	159	27.6	0.532
	Middle	228	39.7	218	37.8	0.554
	High	178	31.0	200	34.7	0.202
First language	English	616	92.1	618	91.3	0.669
	Other language	53	7.9	59	8.7	0.669
Aboriginal status	No	641	95.8	657	97.0	0.284
	Yes	28	4.2	20	3.0	0.284
Pension card status	No	525	78.5	538	79.5	0.704
	Yes	144	21.5	139	20.5	0.704

**Table 3:** Baseline and follow-up ratings by group.

Sub/Scale	Control			Intervention		
	Baseline	Post-test	Chg	Pre-test	Post-test	Chg
ECERS-E	3.09 (0.94)	3.19 (1.12)	+0.10	3.17 (1.03)	4.03 (1.25)	+0.86*
Literacy	3.81 (1.12)	3.79 (1.17)	−0.02	3.89 (1.05)	4.76 (1.21)	+0.87*
Mathematics	2.83 (1.20)	3.24 (1.57)	+0.41	2.87 (1.17)	4.31 (1.66)	+1.44*
Science	3.08 (1.18)	3.19 (1.24)	+0.11	3.19 (1.36)	4.08 (1.64)	+0.89*
Diversity	2.65 (1.02)	2.54 (1.01)	−0.11	2.74 (1.27)	2.99 (1.04)	+0.25
SSTEW	3.96 (1.25)	3.83 (1.28)	−0.13	4.00 (1.21)	4.90 (1.36)	+0.90*
Building T,C,I	4.89 (1.30)	4.47 (1.44)	−0.42	5.03 (1.14)	5.56 (1.25)	+0.53*
Soc-Emo W-B	4.09 (1.70)	4.06 (1.60)	−0.03	4.10 (1.70)	5.15 (1.66)	+1.05*
Lang-Comm	4.44 (1.34)	4.16 (1.53)	−0.28	4.49 (1.24)	5.43 (1.32)	+0.94*
Learn-Critical	2.98 (1.38)	3.03 (1.31)	+0.05	3.08 (1.40)	4.25 (1.61)	+1.06*
Assessing	3.40 (1.48)	3.41 (1.37)	+0.01	3.28 (1.50)	4.10 (1.66)	+0.82*

ECERS-E, average of ECERS-E subscale scores for a given room; SSTEW, average of SSTEW subscale scores for a given room. A score of 1 is considered inadequate, 3 as basic, 5 as good and 7 as excellent quality. Asterisks (*) next to change values denote significant pre-to post-test change according to paired samples t-tests. ECERS-E indicates average change score (baseline to post-intervention) across all ECERS-E subscales. SSTEW indicates average change score across SSTEW subscales. Build TCI, building trust, confidence and independence; SE Wellbg, social–emotional wellbeing; Lang-Comm, supporting and extending language and communication; Learn-Crit, supporting learning and critical thinking. Assessing, assessing learning and language.

### Professional development intervention

The PD programme focused on enhancing the quality of staff interactions and on improving relational and intentional pedagogy with children. The programme, delivered in three phases over 7 months, provided opportunities to observe, discuss, practice and reflect on important attributes of the effective educator’s role, including: engaging in high-quality interactions and sustained, shared thinking (SST), developing and extending concepts and modelling critical and reflective thinking. Links were made to appropriate frameworks, including the Australian NQS and the Australian EYLF. Fundamental to each session was an evidence-based understanding of how young children learn best. The PD was designed to support the collective participation of attendees and to promote collaborative working to gain a deeper knowledge of leadership, change management, quality improvement and self-assessment through two quality rating tools, the ECERS-E and the SSTEW.

The PD content was informed by the pre-assessment of classroom quality measured by ECERS-E and SSTEW, it emphasised relational and intentional pedagogies and high-quality interactions that have demonstrated impact on children’s outcomes (Siraj-Blatchford et al., 2002). Results from the pre-assessment showed that teachers’ performance on literacy, mathematics, science, diversity and supporting critical thinking was on average, minimal quality, thus support for teachers’ knowledge and understanding of effective ECEC pedagogical practices in these aspects. The PD programme focused on eight areas: (1) robust research about quality in ECEC and its assessment; (2) high-quality interactions which extend children’s development; (3) the relevance of self-regulation to children’s educational success; (4) the links between early language development and later literacy; (5) mathematical and scientific concept development in the early years; (6) different ways to use observation, assessment of practice and planning to improve quality; (7) the importance of the early home learning environment and connections across ECEC settings and the home learning environment and (8) the relevance of leadership learning for children’s development—and ways to improve it through the use of self-assessment and planning using the training and the two scales. The PD programme focused on these eight areas to improve teachers’ interaction (process) quality and pedagogical content knowledge that would prepare the teachers for leadership roles within their classrooms and those of their peers. Intentional and relational pedagogies were integrated into these eight areas by introducing the research on effective pedagogy, DVD clips modelling how to relationally and intentionally interact with children to develop their language, mathematics and scientific concepts, and using self-assessment in the PD programme.

The programme was then delivered in three phases at three central hubs. The sessions were conducted by four of the study’s researchers, who are international experts in ECEC. The face-to-face sessions were delivered in a group setting for the centres nearest to each hub.

#### Phase 1: intensive professional development (week 1 to week 3, delivered at three hubs)

A two-day, intensive, face-to-face training provided: an overview of national and international research; an introduction to relevant pedagogical quality characteristics through the use of the ECERS E and SSTEW quality rating scales; coverage of key concepts and ideas; strategies to foster early language, cognitive, self-regulatory and social development; methods of engaging in high-quality interactions and strategies for working with families.

#### Phase 2: follow-up professional development (week 3 to month 3, delivered at three hubs)

Five 4-h, half-day, face-to-face sessions, delivered every 2 weeks, beginning 2 weeks after a hub had completed phase 1. The sessions included time for reflection; planning and critical analysis; an introduction to knowledge and pedagogical content about areas not covered in Phase 1.

#### Phase 3: model for sustainability (week 3 to month 7, delivered online)

To promote centre persistence, limit the effects of staff turnover and increase the likelihood of a positive impact, PD support was provided for the full 7-month intervention through online modules (beginning at the end of phase 1 and continuing for 7 months). Activities and resources for Phase 3 were designed to promote staff engagement and establish an online community of educators. Online modules combined video-streamed PD PowerPoints and content with questions and text, including links to activities and a discussion forum. Staff participation and discussions, moderated and supported by the research team, fed into a learning portfolio, tracking and reflecting how their ideas about pedagogy, children, families and communities changed. Access to this online environment was provided to all centre staff, not only those attending Phases 1 and 2.

### Measures and procedure

Measures were selected to evaluate intervention effects at two levels: the environmental quality fostered by educators which the PD targeted directly and the diverse child outcomes which the PD targeted indirectly *via* changes in educator practice. Primary outcomes at room level were established through ECERS E and SSTEW standardised quality rating scales. A range of child measures were selected to include outcomes important for school readiness (e.g., literacy, numeracy, self-regulation, and social development).

#### Quality ratings

To evaluate the effects of the PD on educators’ classroom practice, quality ratings (using the two established scales with predictive validity; Sylva et al., 2006; Howard et al., 2018) were conducted by highly trained observers through a one-day observation of each preschool room in the participating ECEC centres. All observers were required to achieve a rigorous standard of inter-rater reliability with a highly experienced observer, as indexed by: an intra-class correlation in ratings >0.70 (*M* = 0.86); a mean difference in ratings <0.75 (*M* = 0.43) and at least 80% of item ratings within 1 point (*M* = 93%). In all cases, the researchers involved in collecting baseline and outcome data were blinded to centres’ group allocation.

##### Early childhood environment rating scale-extension

The ECERS-E measures the quality of the curricula, environment and pedagogy in ECEC settings (Sylva et al., 2006). It comprises 15 items which yield four subscales: (1) literacy, (2) mathematics, (3) science and environment, and (4) diversity. Every ECERS-E item is rated from 1 (inadequate practice) to 7 (excellent practice) derived from observers’ on-balance judgments about the presence or absence of the scale’s quality indicators across a one-day room observation. ECERS-E has been shown to have good reliability and predictive validity of child-development progress at school entry (Sylva et al., 2006). Items in each subscale were averaged to create subscale scores. Subscales were averaged to generate an overall scale score.

##### Sustained shared thinking and emotional wellbeing scale

The SSTEW scale brings together different dimensions of the ECEC environment to consider pedagogy which supports children under five in developing skills in sustained shared thinking and emotional wellbeing (Siraj-Blatchford et al., 2015). The scale contains 14 items across five subscales: (1) building trust, (2) confidence and independence, (3) social and emotional wellbeing, (4) supporting and extending language and communication and (5) supporting learning and critical thinking and assessing learning and language. Like the ECERS-E, each scale item is rated from 1 (inadequate) to 7 (excellent) based on the pattern of the presence/absence of the item’s quality indicators. SSTEW has been shown to have good reliability and predictive validity of child development (Howard et al., 2018). Items are averaged to yield subscale scores, and the subscales are averaged to generate an overall scale score.

#### Child assessments

In total, the child outcome measurements involved 40–50 min of direct assessment per child (split into two sessions) and educator social–emotional ratings of the children at both data collection time points. In all cases, a highly trained fieldworker, who was blind to environmental assessments and group assignments conducted child assessments in a quiet area in the child’s ECEC centre. Assessor training involved full-day training on the assessments, expert observation and feedback from the administration and ongoing feedback from regular quality control checks of the data.

##### Language development

The first language assessment—the Verbal Comprehension subtest of the Differential Ability Scales (DAS-II; Elliott, 2007) – comprises 42 items which ask children to identify and manipulate objects in response to verbal instructions. Assessment continues until the earlier completion or non-satisfaction of a performance threshold at identified stop rule junctures. The DAS-II is appropriate for use from 2.5 to 17 years of age, and has shown good reliability (internal consistency, test–retest reliability) and validity (concurrent, predictive) in children within and outside typical development ranges (Elliott, 2007). The second language assessment, the Early Years Toolbox (EYT) Expressive Vocabulary (Howard and Melhuish, 2016), is a 54-item measure of a child’s expressive vocabulary which requires children to produce verbally the correct label for each depicted stimulus. The measure ceases at the earlier of completion or six consecutive incorrect responses. This assessment has been used successfully with children aged 2.5 to 6 years, with good internal consistency and convergent validity in a large and demographically diverse sample (Howard et al., 2018).

##### Numeracy development

The first numeracy assessment, Early Number Concepts subscale of the DAS-II, contains 33 items and requires children to count, identify digits and quantities, perform basic mathematical operations and demonstrate knowledge of basic numerical concepts. Administration rules and assessment properties parallel those for Verbal Comprehension. The Early Number Concepts subscale of the Differential Ability Scales has good internal consistency and concurrent validity (Elliott, 2007). In addition, four Preschool Early Numeracy Scale (PENS) subscales were administered to capture elements of early numeracy not assessed in the DAS-II. These were: one-to-one counting; counting subsets; number order and set-to-numerals. A total of 21 PENS items were administered: PENS is designed for use with children from 3 years of age and has good reliability and predictive validity (Purpura and Lonigan, 2015).

### Analysis strategy

The effects of the PD intervention on quality ratings were analysed by linear regression models, initially across the full sample (i.e. *intention-to-treat*), after controlling for other variables which might also account for observed differences (geography, service type, NQS rating, area-level SES, quality ratings at baseline). To consider the effect of the PD amongst those centres which maintained a minimum threshold of participation (to examine its effect more accurately with adherence), these analyses were repeated with *a per-protocol* sample.

To examine the impact of PD on children’s development outcomes, analyses were conducted that compared the difference between post-and pre-intervention test scores. Initially, this was undertaken using simple uncontrolled comparisons between intervention and control groups. Subsequent comparisons between intervention and control groups controlled for a range of covariates, including child gender, mother’s education, family income, first language status, aboriginal status and receipt of benefits (pension card). The randomised controlled trial involved 1,346 children in 83 centres: 38 centres received the intervention, whilst 45 centres acted as controls. The unit of analysis was children attending the centres.

Mixed-effect linear regression models were used with a random effect fitted for cluster (i.e. childcare centre), which accounted for clustering of repeated assessments within individuals and clustering of individuals within centres. Models were fitted to multiply imputed and complete cases data.

To allow for the fact that there was some missing data in the covariates, multiple imputation was used. Ten multiply imputed data sets were generated using the Amelia package for R. (Honaker et al., 2011; R Core Team, 2020). All outcomes and covariates were included in the multiple imputation model. Models were fitted to the multiply imputed data sets and results were consolidated using Rubin’s (1987) rule, using Hesterberg’s estimate for the degrees of freedom (Hesterberg, 1998).

## Results

### The impacts of the professional development programme on pedagogical quality

#### Descriptive analysis

After the PD programme, significant changes were observed in the intervention group regarding all of the subscales of SSTEW and ECERS-E except diversity, while there were no significant differences in the scores changes in the control group (all *p*s > 0.05). In particular, the scores change in the intervention group ranged from 0.25 to 1.44 (all *p*s < 0.05 except ECERS-E diversity). The scores of ECERS-E mathematics showed the most changes (change = 1.44, *p* < 0.05), followed by SSTEW learning and critical thinking (change score = 1.06, *p* < 0.05). Meanwhile, the SSTEW building trust, confidence and independence showed the least but significant changes (change score = 0.53, *p* < 0.05), followed by SSTEW assessing (change score = 0.82, *p* < 0.05). However, ECERS-E diversity showed the least and non-significant changes (change score = 0.25, *p* > 0.05).

#### Full sample (intention-to-treat) evaluation

Efficacy of the Leadership for Learning intervention for effecting positive change in ECEC quality was evaluated using regression analyses, adjusting for geography, service type, NQS rating, area-level SES and baseline quality ratings, across the full sample. The results indicated a significant effect of the intervention for both scales—ECERS-E: *F*(6,92) = 14.20, *p* < 0.001, *R^2^* = 0.50, SSTEW: *F*(6,92) = 22.23, *p* < 0.001, *R^2^* = 0.61—and subscales (all *p*s < 0.05). As shown below in Table 4, group was a significant predictor for all scales and subscales (*ß*s ranging from.20 to.38). The control variables, except SEIFA and geographic category, also were significant predictors of quality levels in the expected manner (i.e. preschools, higher NQS and higher quality ratings at baseline were each associated with higher post-intervention quality ratings).

**Table 4:** Standardised beta weights for predictors of post-intervention ECERS-E and SSTEW ratings, intention-to-treat.

	ECERS-E					SSTEW					
	Overall	Literacy	Math	Science	Diversity	Overall	Build T,C,I	Soc-Emo	Lang	Lear-Crit	Assessing
	Std. B	Std. B	Std. B	Std. B	Std. B	Std. B	Std. B	Std. B	Std. B	Std. B	Std. B
Intention-to-treat											
Group	0.31*	0.35*	0.29*	0.26*	0.20*	0.35*	0.35*	0.29*	0.38*	0.35*	0.23*
Geog. Cat	0.06	0.08	0.09	−0.01	0.09	0.07	0.08	0.04	0.08	0.07	0.09
Service type	0.26*	0.28*	0.23*	0.19*	0.30*	0.27*	0.30*	0.20*	0.27*	0.25*	0.26*
NQS rating	0.37*	0.31*	0.36*	0.39*	0.27*	0.42*	0.33*	0.47*	0.34*	0.38*	0.32*
SEIFA dec.	0.03	0.12	0.07	−0.02	−0.02	0.12	0.13	0.04	0.14	0.12	0.08
ERS T1	0.29*	0.29*	0.22*	0.23*	0.22*	0.32*	0.13	0.24*	0.25*	0.31*	0.49*
PD attend	0.36*	0.36*	0.35*	0.34*	0.20	0.37*	0.19	0.34*	0.34*	0.45*	0.33*

Initial regressions considered associations of group with subsequent quality, controlling for the complement of covariates. A subsequent regression removed the group variable and, instead, entered a PD attendance variable to investigate the association between level of PD attendance and subsequent quality, after controlling for this same complement of covariates. *p < 0.05; **p < 0.001.

#### Participating sample (per-protocol) evaluation

Given that intention-to-treat analyses provide a generally conservative estimate of the intervention’s effect (Gupta, 2011), subsequent intervention analyses typically consider those who met a sufficient threshold of PD participation and adherence to intervention protocols (a per-protocol evaluation). Per-protocol adherence was referenced against the study’s requirement for at least two staff members from each centre to attend the face-to-face PD. To create an index of a centre’s attendance, two core principles were considered: (1) that no face-to-face session was more important than another (thus, sessions were divided into half-days to provide a uniform metric) and (2) that there is additional benefit from a second (and third, etc.) educator attending the PD, although the degree of benefit is likely diminishing with each additional educator in attendance. As such, attendance was considered using the following formula: [(# of half-days attended by Educator 1) + ([# of half-days attended by Educator 2 * 0.50) + ([# of half-days attended by Educator 3 * 0.33)]. This generated a maximum score of 16.50, representing three educators attending all Phase 1 and Phase 2 sessions.

The mean attendance score for all intervention centres was 12.77 (*SD* = 2.50, range = 5.00–16.50). One centre did not attend Phase 1 at all. All other centres sent at least one educator, with most (86.8%) sending two or more educators. For Phase 2, most centres (84.2%) had at least one educator attend all half-day sessions, four centres (10.5%) had an educator at 4 of the 5 sessions and two centres (5.3%) sent an educator to only 2 of the 5 sessions. Given this pattern of attendance, and stated attendance expectations, the minimum threshold to be included in per-protocol analyses was set at two educators attending the first two full days and at least half the half-days (10.50 points). This threshold removed three intervention centres from per-protocol analyses.

As shown in Table 5, results of the per-protocol regression analyses again indicated a significant effect of the PD intervention for all scales and subscales (*ß*s ranging from 0.22 to 0.40). These effects remained after controlling for identified covariates. The size of the intervention effect, as indicated by standardised regression weights, was improved in nearly all cases (see Table 5).

**Table 5:** Standardised beta weights for predictors of post-intervention ECERS-E and SSTEW ratings, pre-protocol.

	ECERS-E					SSTEW					
	Overall	Literacy	Math	Science	Diversity	Overall	T,C,I	Soc-Emo	Lang	Lear-Crit	Assessing
	Std. B	Std. B	Std. B	Std. B	Std. B	Std. B	Std. B	Std. B	Std. B	Std. B	Std. B
Intention-to-treat											
Group	0.33*	0.37*	0.31*	0.29*	0.22*	0.38*	0.35*	0.32*	0.40*	0.40*	0.27*
Geog. Cat	0.07	0.08	0.11	0.02	0.11	0.07	0.08	0.04	0.09	0.08	0.10
Service type	0.24*	0.27*	0.21*	0.17*	0.28*	0.25*	0.29*	0.18*	0.25*	0.22*	0.24*
NQS rating	0.37*	0.30*	0.36*	0.40*	0.27*	0.41*	0.33*	0.47*	0.34*	0.38*	0.32*
SEIFA dec.	0.05	0.14	0.04	0.01	0.00	0.15*	0.14	0.08	0.16*	0.16*	0.11
ERS T1	0.28*	0.29*	0.22*	0.21*	0.22*	0.36*	0.13	0.28*	0.27*	0.35*	0.52*
PD attend	0.26	0.31	0.27	0.24	0.02	0.17	0.18	0.12	0.16	0.21	0.11

Initial regressions considered associations of group with subsequent quality, controlling for the complement of covariates. A subsequent regression removed the group variable and, instead, entered a PD attendance variable to investigate the association between level of PD attendance and subsequent quality, after controlling for this same complement of covariates. *p < 0.05; **p < 0.001.

### The impacts of professional development on child outcomes

Table 6 shows the initial uncontrolled comparisons between the control and intervention groups for age and the child outcome variables. There were statistically significant pre-post differences between the control and intervention groups for the pre-post age gap, DAS early Number (M *control* = 2.40, M *intervention* = 2.92, *p* < 0.05), DAS verbal comprehension (M *control* = 0.65, M *intervention* = 1.33, p < 0.05) and Preschool Early Numeracy (M *control* = 0.119, M *intervention* = 0.169, *p* < 0.001). However, there was also a significant group difference for the Preschool Early Numeracy pre-test (M *control* = 0.560, M *intervention* = 0.526, p < 0.05). Specifically, the Preschool Early Numeracy scores in the control group were significantly higher than that in the intervention group before intervention.

**Table 6:** Uncontrolled comparisons for age and outcome variables pre-and post-test difference by intervention/control group.

Outcome	Control		Intervention		Value of *p*	N missing	% missing
	Mean	*SD*	Mean	*SD*			
Pre-intervention age	4.592	0.378	4.589	0.361	0.898	0	0.00
Post-intervention age	5.174	0.374	5.177	0.360	0.860	4	0.30
Difference in age gap pre-and post-intervention	0.581	0.052	0.588	0.053	0.012 *	4	0.30
DAS early number pre-intervention	19.90	4.84	19.35	5.08	0.054	127	9.44
DAS early number post-intervention	22.30	4.65	22.27	4.83	0.932	126	9.36
DAS early number pre-post difference	2.40	4.24	2.92	4.11	0.037 *	212	15.75
DAS verbal comprehension pre-intervention	20.50	4.75	20.21	4.90	0.285	127	9.44
DAS verbal comprehension post-intervention	21.20	4.73	21.63	4.96	0.124	132	9.81
DAS verbal comprehension pre-post difference	0.65	5.12	1.33	5.00	0.025 *	216	16.05
EYT expressive vocabulary pre-intervention	27.72	6.81	27.70	6.91	0.953	146	10.85
EYT expressive vocabulary post-intervention	31.00	6.43	31.18	6.39	0.624	126	9.36
EYT expressive vocabulary pre- and post difference	3.27	3.90	3.29	3.60	0.916	228	16.94
Preschool early numeracy pre-intervention	0.560	0.252	0.526	0.269	0.023 *	126	9.36
Preschool early numeracy post-intervention	0.682	0.214	0.680	0.216	0.849	134	9.96
Preschool early numeracy pre- and post difference	0.119	0.173	0.152	0.169	0.001 **	217	16.12

Statically significant t-test value of ps comparing control and intervention groups: *p < 0.05, **p < 0.01.

While there were no significant differences between the groups in demographic characteristics, there is the possibility that the demographic characteristics of the groups may influence the comparisons between the control and intervention groups. Also, as there was a slight difference in the pre-to post-test age gap for the groups, this was also included as a covariate. Hence to be prudent, analyses were undertaken controlling for these covariates. This was done using mixed-effect linear regression models with a random effect for cluster (i.e. childcare centre), to adjust for any effects of such clustering of children. Models were fitted to multiply imputed and complete case data. The results are shown in Tables 6–9.

**Table 7:** Results of the regression model of difference between pre-and post-intervention DAS early number concepts scores; models fitted to multiply imputed data (*N* = 1,134) and to complete cases only (*N* = 961).

		Imputed data			Complete cases		
		Beta	95% CI	Value of *p*	Beta	95% CI	Value of *p*
Group	Control	Reference level			Reference level		
	Intervention	+0.360	+0.208	+0.208	+0.208	(−0.551,+0.966)	0.587
Age difference		+4.862	(−1.345,+11.070)	0.125	+5.793	(−0.929,+12.514)	0.091
Sex	Male	Reference level			Reference level		
	Female	−0.580	(−1.058,-0.101)	0.018*	−0.549	(−1.069,-0.029)	0.038*
Mother’s education	Less than high school	Reference level			Reference level		
	High school	+0.392	(−0.492,+1.276)	0.384	+0.353	(−0.574,+1.280)	0.455
	Diploma	+0.310	(−0.664,+1.285)	0.532	+0.364	(−0.660,+1.389)	0.485
	University or higher	+0.427	(−0.472,+1.326)	0.351	+0.457	(−0.484,+1.398)	0.341
Income band	Low	Reference level			Reference level		
	Middle	+0.030	(−0.645,+0.704)	0.930	+0.023	(−0.657,+0.703)	0.947
	High	+1.032	(+0.329,+1.736)	0.004 **	+1.193	(+0.450,+1.937)	0.002 **
First language	English	Reference level			Reference level		
	Other language	+0.967	(+0.021,+1.913)	0.045 *	+0.920	(−0.073,+1.913)	0.069
Aboriginal status	No	Reference level			Reference level		
	Yes	−0.268	(−1.601,+1.065)	0.693	−0.164	(−1.577,+1.250)	0.820

CI, confidence interval; Statically significant value of ps: *p < 0.05, **p < 0.01.

**Table 8:** Results of regression model of difference between pre-and post-intervention Preschool Early Numeracy Scale scores; models fitted to multiply imputed data (*N* = 1,129) and to complete cases only (*N* = 958).

		Imputed data			Complete cases		
		Beta	95% CI	Value of p	Beta	95% CI	Value of p
Group	Control	Reference level			Reference level		
	Intervention	+0.035	(+0.008,+0.061)	0.011 *	+0.045	(+0.017,+0.073)	0.002 **
Age difference		+0.271	(+0.031,+0.510)	0.027 *	+0.266	(+0.009,+0.523)	0.042 *
Sex	Male	Reference level			Reference level		
	Female	−0.009	(−0.029,+0.011)	0.397	−0.011	(−0.033,+0.010)	0.303
Mother’s education	Less than high school	Reference level			Reference level		
	High school	−0.016	(−0.053,+0.020)	0.385	−0.016	(−0.055,+0.022)	0.406
	Diploma	−0.027	(−0.067,+0.014)	0.203	−0.028	(−0.071,+0.015)	0.206
	University or higher	−0.041	(−0.078,-0.004)	0.032 *	−0.038	(−0.077,+0.001)	0.059
Income band	Low	Reference level			Reference level		
	Middle	−0.009	(−0.038,+0.020)	0.554	−0.006	(−0.034,+0.022)	0.680
	High	−0.016	(−0.046,+0.015)	0.306	−0.014	(−0.046,+0.017)	0.359
First language	English	Reference level			Reference level		
	Other language	−0.011	(−0.050,+0.029)	0.602	−0.013	(−0.055,+0.029)	0.535
Aboriginal status	No	Reference level			Reference level		
	Yes	+0.012	(−0.044,+0.067)	0.676	+0.023	(−0.036,+0.082)	0.451

CI, confidence interval; Statically significant value of ps: *p < 0.05, **p < 0.01.

**Table 9:** Results of the regression model of difference between pre-and post-intervention DAS verbal comprehension scores; models fitted to multiply imputed data (*N* = 1,130) and to complete cases only (*N* = 960).

		Imputed data			Complete cases		
		Beta	95% CI	Value of *p*	Beta	95% CI	Value of *p*
Group	Control	Reference level			Reference level		
	Intervention	+0.673	(−0.113,+1.460)	0.092	+0.618	(−0.241,+1.477)	0.156
Age difference		+1.483	(−5.585,+8.551)	0.681	+2.279	(−5.500,+10.058)	0.565
Sex	Male	Reference level			Reference level		
	Female	−0.329	(−0.919,+0.261)	0.274	−0.535	(−1.172,+0.103)	0.100
Mother’s education	Less than high school	Reference level			Reference level		
	High school	+0.274	(−0.800,+1.349)	0.616	+0.149	(−0.985,+1.283)	0.797
	Diploma	−0.392	(−1.572,+0.788)	0.515	−0.541	(−1.795,+0.712)	0.397
	University/ higher	+0.076	(−1.022,+1.173)	0.893	+0.132	(−1.019,+1.283)	0.822
Income band	Low	Reference level			Reference level		
	Middle	−0.452	(−1.258,+0.354)	0.272	−0.463	(−1.296,+0.370)	0.276
	High	−0.848	(−1.704,+0.009)	0.052	−0.891	(−1.802,+0.020)	0.055
First language	English	Reference level			Reference level		
	Other language	+0.345	(−0.829,+1.518)	0.565	+0.452	(−0.777,+1.681)	0.471
Aboriginal status	No	Reference level			Reference level		
	Yes	−0.361	(−2.002,+1.279)	0.666	−0.328	(−2.061,+1.404)	0.710

CI, confidence interval.

#### Early number concepts scores

As shown in Table 7, age, mother’s education and aboriginal status did not influence the comparisons between the control and intervention groups regarding children’s number concepts. However, gender (ß = −0.580, *p* < 0.05, 95% CI (−1.058,-0.101)), income band (ß = 1.032, *p* < 0.05, 95% CI (0.021, +1.913)) and first language (ß = 0.967, *p* < 0.05, 95% CI (0.021,+1.913)) showed significant influences in the imputed data. In particular, boys, children from high-income families and children whose first language is not English, received higher number concepts scores after their teachers participated in PD intervention.

#### Early numeracy scale scores

As shown in Table 8, gender, income band, first language and aboriginal status did not influence the comparisons between the control and intervention groups regarding children’s number concepts. However, group (ß = 0.035, *p* < 0.05, 95% CI (0.008, 0.061)), age (ß = 0.271, *p* < 0.05, 95% CI (0.031, 0.510)) and mother’s education (ß = −0.041, *p* < 0.05, 95% CI (−0.078, −0.004)) showed significant influences in the imputed data. In particular, children from the control group, younger children and children whose mothers had university or higher education tended to receive lower early numeracy scores in the PD programme.

#### Verbal comprehension scores

As shown in Table 9, age, gender, mother’s education, income band, first language and aboriginal status did not influence the comparisons between the control and intervention groups regarding children’s Verbal Comprehension.

#### Expressive vocabulary scores

As shown in Table 10, age, gender, mother’s education, income band, first language and aboriginal status did not influence the comparisons between the control and intervention groups regarding children’s Verbal Comprehension.

**Table 10:** Results of the regression model of difference between pre-and post-intervention EYT expressive vocabulary scores; models fitted to multiply imputed data (*N* = 1,118) and to complete cases only (*N* = 945).

		Imputed data			Complete cases		
		Beta	95% CI	Value of *p*	Beta	95% CI	Value of *p*
Group	Control	Reference level			Reference level		
	Intervention	+0.099	(−0.499,+0.697)	0.743	+0.064	(−0.595,+0.723)	0.847
Age difference		+4.856	(−0.477,+10.189)	0.074	+3.490	(−2.440,+9.420)	0.248
Sex	Male	Reference level			Reference level		
	Female	+0.068	(−0.370,+0.506)	0.760	+0.166	(−0.315,+0.646)	0.499
Mother’s education	Less than high school	Reference level			Reference level		
	High school	−0.062	(−0.894,+0.769)	0.883	−0.250	(−1.108,+0.609)	0.568
	Diploma	−0.681	(−1.580,+0.217)	0.137	−0.699	(−1.643,+0.245)	0.146
	University or higher	−0.088	(−0.928,+0.753)	0.838	−0.227	(−1.097,+0.643)	0.609
Income band	Low	Reference level			Reference level		
	Middle	+0.405	(−0.215,+1.025)	0.200	+0.493	(−0.134,+1.119)	0.123
	High	−0.236	(−0.897,+0.426)	0.484	−0.235	(−0.921,+0.451)	0.502
First language	English	Reference level			Reference level		
	Other language	+0.057	(−0.822,+0.937)	0.898	−0.148	(−1.079,+0.782)	0.754
Aboriginal status	No	Reference level			Reference level		
	Yes	+0.229	(−1.015,+1.473)	0.718	+0.479	(−0.853,+1.812)	0.480

CI, confidence interval.

## Discussion

Following the theory behind RCTs, and hence assuming that control and intervention groups are equivalent because of randomisation, uncontrolled comparisons should suffice for testing the effect of the intervention. In terms of pedagogical quality, the results from the regression analysis indicated that *Leadership for Learning* PD showed significant effects on the total average and subscale scores of SSTEW and ECERS-E, the latter was also part of the PD. In terms of child development, in simple uncontrolled comparisons between control and intervention group children, there were significantly greater improvements in the intervention group for the change between pre-and post-test for the outcomes DAS Early Number Concepts, DAS Verbal Comprehension and Preschool Early Numeracy Scale. However, in the mixed-effect linear regression model, only the Preschool Early Numeracy showed a significant difference indicating greater improvement for the intervention group. Meanwhile, children’s age, gender, family income, first language and mother’s education significantly influence the PD impacts on children’s number concept and numeracy development. These findings are discussed in this section.

### The impacts of the professional development programme on pedagogical quality

The current research identified that the *Leadership for Learning* PD had effects on the curricular and interactional quality measured by ECERS-E and SSTEW. The average improvement for ECERS-E and SSTEW were 0.86 and 0.90 on seven-point scales. This result aligns with recent meta-analyses, indicating that in-service PD programmes could promote classroom quality and teacher-child interaction (Egert et al., 2018), especially when staff can use them for self-assessment, planning and improvement too.

The components of this PD programme could explain the significant effects. Regarding the “what” component of the *Leadership for Learning* PD, our results suggest that integrating relational and intentional pedagogy in a child-development-oriented approach effectively improves teachers’ pedagogical practice. More specifically, as previously stated, the PD content involves pedagogical knowledge and strategies to foster relational and intentional educators. Meanwhile, these are not general pedagogical knowledge or strategies but have been tailored to children’s cognitive and social–emotional development in different PD sessions, such as literacy, mathematical and scientific concept development and self-regulation. This is, to some extent, consistent with previous research, which identified training teaching strategies according to the discipline-specific curriculum (Buysse et al., 2009; Darling-Hammond et al., 2017). Meanwhile, the *Leadership for Learning* PD also extends this statement by replacing discipline-specific content with a child-development-oriented approach as the interdisciplinary curriculum is advocated in preschools to help children gain a more holistic approach to knowledge (Jacobs, 1989; Hall and Pais, 2021). Therefore, the PD content contributes to the discussions on PD design for preschools or contexts without discipline-specific curricula.

Regarding the “how” component of the *Leadership for Learning* PD. The *Leadership for Learning* PD employed face-to-face training, on-site modelling, providing DVD exemplars and reading materials, and phased process evaluations (Siraj et al., 2018). For one thing, the results imply that focusing on practice in the PD through modelling and providing exemplars is a critical element of effective PD. Especially for PD programmes focusing on pedagogical practice, modelling and exemplars can help teachers positively implement higher quality teacher-child interactions. In line with this, evidence reviewed in previous research also suggests that teachers’ practice is more important than PD duration, as PD duration is not necessarily associated with teachers’ practice (Sims and Fletcher-Wood, 2021). This research extends the discourse on effective PD by employing new elements—using quality rating scales and phased process evaluation by teachers themselves. Specifically, teachers learned about elements of the quality ratings of classroom quality and were invited to evaluate the PD provision and their learning process after each phase of PD. Using scales as tools to improve quality has increasingly been applied to research and practice and led to quantifiable improvements in ECEC by democratising the use of these scales by teachers to support their own pedagogical leadership practice (Mathers et al., 2007). The current study integrated this approach into the Leadership for Learning PD and demonstrated its effectiveness. Supporting teachers’ independent use of self-rating using items from the scales supported their practice-uplift. They also evaluated the PD process and provided feedback that informed the follow-up PD design. These approaches support the self-directed learning theory for adult learners, which indicates that adults have a high level of ownership over their own learning, such as self-assessment, setting their learning goals and choosing learning activities (Lohman and Woolf, 2001). Therefore, adapting PD programmes towards teachers’ needs and interests might also increase PD effectiveness in this research.

Regarding the “who” component of the *Leadership for Learning* PD. The PD emphasises the Australian ECEC context and maximises the coherence of PD content with the governmental regulations and national standards of quality ECEC in Australia. The critical role of coherence in designing effective PD programmes has also been emphasised by Desimone (2009), who argues that PD “should be aligned with state and district goals” (p. 184). Our results provided empirical evidence for this statement by examining the effectiveness of a context-fit PD programme. This is especially important for pedagogical practice since different social cultures and contexts have different expectations of teachers’ behaviours (Desimone, 2009). In addition to the context, as aforementioned, teachers’ feedback also informed this PD design. In this regard, teachers are active participants rather than merely passive recipients in this PD programme, thus enabling the match between the PD receivers’ needs and PD provision (Louws et al., 2017). The PD was also modified prior to delivery given the pre-intervention results of the ECERS E and SSTEW, ascertaining where weaknesses existed in staff knowledge and pedagogical approaches.

### The impacts of the professional development programme on child development

RCTs ensure control and intervention groups are equivalent because of randomisation (Torgerson and Torgerson, 2012). Therefore, uncontrolled comparisons should suffice for testing the effect of the intervention, which indicated that there were significantly greater improvements in the intervention group regarding DAS Early Number Concepts, DAS Verbal Comprehension and Preschool Early Numeracy Scale. These results are in line with previous research, indicating that evidence-based in-service professional development could promote children’s language and numeracy development (e.g., Fukkink and Lont, 2007; Markussen-Brown et al., 2017; Egert et al., 2018; Kraft et al., 2018) and that quality pedagogical practice is associated with child development (e.g., Sylva et al., 2006; Werner et al., 2015; McCoy and Wolf, 2018).

Furthermore, mixed-effect linear regression models controlling for clustering and all demographics, including age were used to look for differences in change in outcomes between the control and intervention groups. In these analyses, only the Preschool Early Numeracy showed a significant difference indicating greater improvement for the intervention group. However, the analyses of all the other child outcomes indicated a greater improvement in the intervention group that did not reach statistical significance. For one thing, given that ECERS-E mathematics showed the most dramatic improvement than other subscales, the significant Preschool Early Numeracy scores might be related to the biggest changes in ECERS-E mathematics. Pianta et al. (2021) also observed that teachers performed better improvements in instructional interactions if they had more engagement in coaching feedback. Meanwhile, more gains predicted greater increases in child development. This research and Pianta et al. (2021) finding suggests there might be a threshold between teachers’ amount of pedagogical improvement and facilitating child development, thus requiring future research.

Also, there was some drop-out of centres from the intervention group after randomisation. While the comparisons of demographics indicate no significant differences between the groups, it might still be possible that some differences may exist. Therefore, the study further explored the influencing factors of the PD impacts on child development by analysing the covariates. The findings are discussed in the following section.

### The influencing factors of professional development impacts on child development

In terms of the Early Number Concepts, children’s gender, family income and first language are significant covariates. First, it was noticeable that the difference between pre-and post-intervention scores was significantly smaller for girls than boys. This may represent a degree of “catch up” in boys’ scores over time. Previous research indicated that teacher-child interactions are gendered, and boys tend to receive more negative feedback from teachers than girls, thus influencing their development (Beaman et al., 2006). In this regard, PD programmes, which have been demonstrated to be effective in changing teachers’ response patterns to boys and girls (Consuegra and Engels, 2016), might explain boys’ better gains than girls.

Second, the difference between pre-and post-intervention DAS Early Number Concepts scores was significantly larger for children from high-income families, as compared to the low-income reference group. Although the PD intervention has been demonstrated a buffering role in child development from disadvantaged backgrounds, the development gap could still exist if higher-income children’s teachers also receive training. The result is supported by a recent large-scale longitudinal research indicating that wealth is associated with children’s academic and behavioural development (Miller et al., 2021). Third, children whose first language was not English had a significantly larger difference between pre-and post-intervention DAS Early Number Concepts scores than children whose first language was English. However, this effect was significant in the multiply imputed data model only, and it is prudent to place greater confidence in results supported by both analyses of imputed and complete cases data.

In terms of Preschool Early Numeracy Scale scores, there was a significantly larger improvement between pre-and post-intervention Preschool Early Numeracy Scale scores in the intervention group as compared to the control group, and this difference was larger where the age gap pre-to post-intervention was larger. This might reflect the longer exposure time of children to intervention-trained staff. Additionally, the difference between pre-and post-intervention Preschool Early Numeracy Scale scores was significantly smaller for children whose mothers were educated at university or higher level as compared to children whose mothers had educational attainment of “less than high school.” This may represent a degree of “catch up” over time in the scores of the children of lower qualified mothers. Although mothers’ education provides a foundation that supports children’s academic success (Davis-Kean et al., 2021), the results of this study contribute to current research on parental education by showing that providing PD programmes for teachers could partially compensate for the negative influence on child development from the relatively lower levels of mothers’ education.

In terms of DAS Verbal Comprehension and the EYT Expressive Vocabulary, there were no statistically significant effects in the regression models for their outcomes. Our result suggests that the covariates of PD programmes might have different mechanisms of influencing mathematics and language development. Chow and Ekholm (2019) also identified that vocabulary did not significantly predict mathematics. Therefore, some covariates which are significantly related to child numeracy might not be associated with child language.

The discussion has focused mainly on teacher quality and child cognitive outcomes. There were expected improvements in children’s socio-behavioural and self-regulation outcomes from pre-to post-test in the control group for children in routine ECEC practice. By contrast, children in the intervention group showed an additional improvement over the same period, but only for internalising problems. The intervention did not appear to produce an added benefit for children in the intervention group in relation to externalising problems, prosocial behaviours and self-regulation (for further insight please see the technical report, Siraj et al., 2018).

## Conclusion

This study examines the effects of an evidence-informed PD, the *Leadership for Learning* training which contains curricula content, process quality and child-development workshops. The PD was predicated on 4 important principles: (1) Reviewing the extant literature and meta-analyses of “what works” in PD eg best duration, specific knowledge of child development as well as content, delivery and modes of presentation, (2) use of pre-assessment quality rating scales data from the study to identify specific training needs, (3) use of the scales as tools for the teachers to support their learning and practice, alongside workshop and supporting materials, and (4) supporting staff by using their feedback through questionnaires alongside online ongoing support with access to all materials and a platform for a community of learners.

The results indicated that *Leadership for Learning* PD significantly affected pedagogical quality, with the intervention group receiving higher scores of SSTEW and ECERS-E as expected, given their use as assessment and support. In terms of child development, children in the intervention group also showed significantly greater improvements in socio-emotional, numeracy and language skills measured by DAS Early Number Concepts, DAS Verbal Comprehension and Preschool Early Numeracy Scale. Children’s age, gender, family income, first language and mother’s education significantly influence the PD impacts on children’s number concept and numeracy development. Within a fragmented system of ECEC with variable training of the workforce, expanding access to ECEC and improving quality by providing effective PD can promote children’s outcomes and may contribute to school readiness for this age group.

Future research on PD intervention could consider the following areas. Firstly, the participants of the current study were from Australia, thus warranting caution in terms of generalising the research findings to other contexts. Given that teachers in different contexts show variability in pedagogical practice and PD needs, PD adaptation is required when conducting the Leadership for Learning PD in other contexts. Secondly, this research focused on classroom quality and child development and did not collect data on the home learning environment and teachers’ characteristics that also impact child development. Future research could control the home learning environment and teachers’ beliefs, self-efficacy and leadership as covariates when exploring the PD effects.

## Data availability statement

The raw data supporting the conclusions of this article will be made available by the authors, without undue reservation.

## Ethics statement

The studies involving human participants were reviewed and approved by the University of Wollongong Ethics Committee, NSW, Australia. Written informed consent to participate in this study was provided by the participants’ legal guardian/next of kin.

## Author contributions

IS, EM, DK, SH, and CN-H contributed to conception and design of the study. IS and SH wrote the first draft of the manuscript. EM, SH, IS, and JG analysed the data. MD, DK, CN-H, RH, and BL read and edited sections of the manuscript. All authors contributed to the article and approved the submitted version.

## Funding

This study was supported by the NSW, Australia, Department of Education and Communities (RFT DECEAR-15-35).

## Conflict of interest

The authors declare that the research was conducted in the absence of any commercial or financial relationships that could be construed as a potential conflict of interest.

## Publisher’s note

All claims expressed in this article are solely those of the authors and do not necessarily represent those of their affiliated organizations, or those of the publisher, the editors and the reviewers. Any product that may be evaluated in this article, or claim that may be made by its manufacturer, is not guaranteed or endorsed by the publisher.
